# Sequence Based Prediction of DNA-Binding Proteins Based on Hybrid Feature Selection Using Random Forest and Gaussian Naïve Bayes

**DOI:** 10.1371/journal.pone.0086703

**Published:** 2014-01-24

**Authors:** Wangchao Lou, Xiaoqing Wang, Fan Chen, Yixiao Chen, Bo Jiang, Hua Zhang

**Affiliations:** School of Computer and Information Engineering, Zhejiang Gongshang University, Hangzhou, PR China; University of South Florida College of Medicine, United States of America

## Abstract

Developing an efficient method for determination of the DNA-binding proteins, due to their vital roles in gene regulation, is becoming highly desired since it would be invaluable to advance our understanding of protein functions. In this study, we proposed a new method for the prediction of the DNA-binding proteins, by performing the feature rank using random forest and the wrapper-based feature selection using forward best-first search strategy. The features comprise information from primary sequence, predicted secondary structure, predicted relative solvent accessibility, and position specific scoring matrix. The proposed method, called DBPPred, used Gaussian naïve Bayes as the underlying classifier since it outperformed five other classifiers, including decision tree, logistic regression, k-nearest neighbor, support vector machine with polynomial kernel, and support vector machine with radial basis function. As a result, the proposed DBPPred yields the highest average accuracy of 0.791 and average MCC of 0.583 according to the five-fold cross validation with ten runs on the training benchmark dataset PDB594. Subsequently, blind tests on the independent dataset PDB186 by the proposed model trained on the entire PDB594 dataset and by other five existing methods (including iDNA-Prot, DNA-Prot, DNAbinder, DNABIND and DBD-Threader) were performed, resulting in that the proposed DBPPred yielded the highest accuracy of 0.769, MCC of 0.538, and AUC of 0.790. The independent tests performed by the proposed DBPPred on completely a large non-DNA binding protein dataset and two RNA binding protein datasets also showed improved or comparable quality when compared with the relevant prediction methods. Moreover, we observed that majority of the selected features by the proposed method are statistically significantly different between the mean feature values of the DNA-binding and the non DNA-binding proteins. All of the experimental results indicate that the proposed DBPPred can be an alternative perspective predictor for large-scale determination of DNA-binding proteins.

## Introduction

DNA-binding proteins play key roles in a wide variety of molecular functions, including recognizing specific nucleotide sequences, maintenance of cellular DNA, transcriptional and translational regulation, DNA replication, and DNA damage repair [1]–[3]. Currently, both computational and experimental techniques have been developed to identify the protein-DNA interactions. The experimental techniques such as filter binding assays [4], ChIP-chip [5], genetic analysis [6] and X-ray crystallography [7] can provide a detailed picture about the binding, however, they are both time-consuming and expensive [3]. Thus, it is highly desired to develop automated computational methods for identifying the DNA-binding proteins from the extremely fast increased amount of newly discovered proteins [8].

So far, a number of predictors of DNA-binding proteins have been proposed. These methods can be divided into two categories, structure based modeling [9]–[18] and sequence based prediction [8], [19]–[30]. Since the protein structure could directly reveal its function mechanics, the availability of structure information about a given protein is believed to contribute towards predicting its function and to provide higher performance than sequence based methods. However, the pitfall of various structure-based methods for predicting DNA-binding function is that they are all limited to a relatively small number of proteins for which high-resolution three-dimensional structures are available. In a contrast, sequence based methods have the main advantage with no need for known structures and thus can be applied to large-scale datasets and genomics targets. For instance, Szilágyi and Skolnick [24] used logistic regression to predict the DNA-binding proteins from the amino acid composition. Kumar *et al*. [23] utilized support vector machine and coded the features from evolutionary profiles for the prediction of DNA-binding proteins. Another group, Kumar *et al*. [22], proposed DNA-Prot method for the classification of the DNA-binding proteins using random forest. Gao and Skolnick [19] proposed a threading-based method which required only the target protein sequence to identify the DNA-binding domains based on a template library composed of DNA-protein complex structure. Lin *et al.* [8] developed a DNA-binding protein predictor using random forest by integrating the features into the general form of pseudo amino acid composition with grey model. The latest work by Zou *et al.* [20] provided a comprehensive feature analysis using support vector machine for the prediction of DNA-binding proteins. As a summary, sequence based prediction methods for DNA-binding proteins have been investigated with several classifiers such as logistic regression [24], random forest [8], [22], support vector machine [20], [21], [23], [25]–[30], and threading based method [19], using various features including (pseudo) amino acid composition [8], [20], [22]–[30], physicochemical properties [20]–[22], [28], predicted secondary structure [20], [22], [28], predicted solvent accessibility [28], evolutionary profile [20], [23], and their various transformations.

The aim of this work is to propose a new predictor for determination of the DNA-binding proteins based on the features composed of sequence, predicted solvent accessibility, predicted secondary structure, and evolutionary profiles. The size of the feature set was reduced by ranking the features using random forest and furthermore by a wrapper based feature selection using best-first forward search strategy based on Gaussian naïve Bayes. The differences between this work and the previous studies are reflected mainly in four aspects: (1) we designed new features concerning the hybrid forms of the amino acid composition, predicted solvent accessibility and predicted secondary structure, the auto-correlation coefficients of the position specific scoring matrix (PSSM), and the percentile values of PSSM scores; (2) we applied random forest to rank the feature importance and subsequently performed wrapper based feature selection based on best-first forward search strategy; (3) we compared the prediction performance of several classifiers including Gaussian naïve Bayes, logistic regression, decision tree, random forest, k-nearest neighbor and support vector machines under the proposed framework and found that Gaussian naïve Bayes outperformed other considered machine learning methods; (4) we conducted a much more complete fair comparison of the proposed model tested on several different independent datasets with the existing sequence-based methods that have web server or standalone software version, which include iDNA-Prot [8], DNA-Prot [22], DNAbinder [23], DNABind [24] and DBD-Threader [19], to our best knowledge. The results show that the proposed method, called DBPPred, is an improved and alternative method for identifying the DNA-binding proteins.

## Materials and Methods

### 2.1. Datasets

DNA-binding protein sequences comprising the training dataset and the independent dataset were extracted from Protein Data Bank (PDB) [31] by searching the mmCIF keyword of ‘DNA binding protein’ through the advanced search interface. The entire set after removing the chains with length of less than 60 and character of ‘X’ was subsequently clustered with NCBI’s BLASTCLUST [32] at 25% sequence identity. A dataset, called PDB390, was created by selecting one chain in each cluster and was finally composed of 390 protein chains with local 25% pairwise sequence identity. Furthermore, DBP390 was divided into two datasets, the training dataset in which the sequences were deposited in PDB before Jan, 2011 and the remaining independent dataset. As a result, the training dataset, named as DBP297, is composed of 297 protein chains, and the independent set, called DBP93, comprises 93 protein chains deposited in PDB after Jan, 2011. Such division based on the deposition date is to avoid the sequence intersection and similarity as much as possible with the training sets used in the existing methods including iDNA-Prot [8], DNA-Prot [22], DNABinder [23], DNABind [24] and DBD-Threader [19], since these methods were published before or in 2011. Thus the blind test can be performed on the independent dataset for a relatively fair comparison with the existing methods.

Similarly, 390 non DNA-binding proteins were randomly selected from a set that was deposited in PDB between Jan, 2011 and Dec, 2012 and was clustered with BLASTCLUST [32] at 25% sequence identity. The set was furthermore divided into two datasets based on the deposition dates of the sequences. These two sets are respectively called NDBP297 composed of 297 chains for training and NDBP93 consisting of 93 chains for independent blind test, where the deposition dates of the sequences in NDBP93 are newer than NDBP297. Accordingly, the benchmark dataset, called PDB594, consists of 594 chains by combining DBP297 and NDBP297, and the independent set, named as PDB186, comprises 186 chains by merging DBP93 and NDBP93. The PDB IDs of PDB594 and PDB186 together with the information concerning primary sequence and deposition date in PDB are listed in Dataset S1 and Dataset S2, respectively.

The small number of DNA-binding proteins as compared to the enormous number of proteins deposited in PDB demonstrates that DNA-binding proteins are only a fraction of all proteins. We collected an independent set composed of a few hundreds of totally non-DNA binding proteins, in order to investigate the false positive rates of the proposed work and the relevant existing methods. This set includes sequences that were deposited in PDB between Jan. 2011 and Nov. 2013 and that contain no DNA binding proteins and no ‘X’ characters. Next, BLASTCLUST with the local identity threshold at 25% was applied to the union of this set, PDB594 and PDB186. The independent non-DNA binding protein set was constructed by selecting one chain with length >60 from each cluster that contains no sequences from PDB594 and PDB186. Consequently, this dataset, called NDBP4025, includes 4025 non-DNA binding proteins that have local identity of at most 25% with each other and also with the protein chains from PDB594 and PDB186.

Moreover, another similar issue, i.e. the prediction of RNA-binding proteins, has been focused on by recent several studies [33]–[36]. We examined the ability of the proposed method and several other existing predictors to distinguish RNA and DNA binding proteins. Two datasets including only RNA-binding proteins, RB-C174 and RB-IC257 used in [34], were used to test the ability for separating DNA and RNA-binding proteins. One sequence in RB-IC257 was removed since it contains ‘X’ characters. These two datasets are renamed RB174 and RB256, which include 174 and 256 RNA-binding proteins, respectively, and their union is denoted by RB430. The RB430 dataset includes 430 sequences that have local identity of at most 25% with each other described in [34]. Similarly as NDBP4025, the sequences in RB430 should be regarded as non-DNA binding proteins, which are examined to compute the false positive rates of considered methods.

### 2.2. Features

One of the steps for designing predictor is to convert the input protein sequence into a set of numerical features that are fed into the classifier to generate prediction of the DNA-binding proteins. The features in this study are coded from primary sequence, predicted secondary structure (PredSS), predicted relative solvent accessibility (PredRSA), position specific scoring matrix (PSSM) generated by PSI-BLAST [32]. They are divided into four categories, secondary structure based, average RSA based, amino acid (AA) composition based, and PSSM score based (see Table 1). The raw features concerning PredRSA and PredSS are derived by SPINE-X program [37], which was evaluated with high quality outcomes for predicting secondary structures and RSA values. Meanwhile, SPINE-X provides the PSSM outputs generated by PSI-BLAST [32].

**Table 1:** Summary of the considered features, where *x*, *x*′ = {A, R, N, D, C, Q, E, G, H, I, L, K, M, F, P, S, T, W, Y, V} denotes the 20 AA types, *y* =  {C, H, E} denotes the three secondary structure states, *h* = {0.1, 0.2, 0.3, 0.4, 0.5} denotes the cutoff used to categorize the buried/exposed residues based on their relative solvent accessibility, *t* = {0, 25, 50, 75, 100} denotes the ratio for computing the percentile values, and *m* = {1, 2, 3, 4, 5, 6, 7, 8, 9, 10} denotes the lag for calculating the auto-correlation coefficients.

Category	Feature description	Abbreviation	No. of features
SS based	Content of the residues with secondary structure type *y*	Con_SS*_y_*	3
Average RSA based	Average RSA of the residues with AA type *x*	AveRSA_Res*_x_*	20
	Average RSA of the residues with secondary structure type y	AveRSA_SS*_y_*	3
	Average RSA of the residues with AA type x and secondary structure type y	AveRSA_Res*_x_*_SS*_y_*	60
Amino acid composition based	Composition of the residues with AA type *x*	AAC_Res*_x_*	20
	Composition of the residues with AA type *x* and secondary structure type *y*	AAC_Res*_x_*_SS*_y_*	60
	Composition of the residues with AA type *x* and RSA value≥*h* (i.e., the residueis assumed exposed)	AAC_Res*_x_*_Ex*_h_*	100
	Composition of the residues with AA type *x* and RSA value<h (i.e., the residueis assumed buried)	AAC_Res*_x_*_Bu*_h_*	100
	Composition of dipeptide with the left AA type *x* and right AA type *x*′	DIC_Res*_xx_*_′_	400
PSSM score based	Average PSSM score of the residues along with the column of amino acid type *x*	AvePscore_AA*_x_*	20
	Average PSSM score of the residues with AA type *x*′ along with the column ofamino acid *x* in the PSSM matrix	AvePscore_AA*_x_*_Res*_x_*_′_	400
	Percentile of the PSSM scores according to the percent threshold *t* along withthe column of amino acid *x*	Pscore_AA*_x_*_P*_t_*	100
	Auto-correlation coefficient of scores with lag *m* along with the column ofamino acid *x*	AutoCC_AA*_x_*_Lag*_m_*	200

The motivation for using PredSS comes from several studies that have shown the benefit to the protein function predictions, including protein folding rate [38] and kinetic type [39], binding residues [40] and catalytic sites [41]. SPINE-X predicts three types of secondary structures, i.e. helix (H), strand (E), and coil (C). The SS based features are coded by the secondary structure content in total number of 3.

The relative solvent accessibility (RSA) is defined as the solvent accessible surface area (ASA) of a given residue normalized by the ASA of this residue in an extended tripeptide, Ala-X-Ala, conformation [42]. The RSA values are often used to distinguish between the interior and the surface of proteins by setting a cutoff. For a given cutoff *h*, the residue with RSA≥*h* are considered to be solvent exposed; otherwise, they are assumed to be buried. We followed our previous work [39] for the determination of protein folding kinetic types and computed average RSA (AveRSA) values over the residues with certain AA type, with a given predicted secondary structure conformation, and with certain AA type and predicted secondary structure conformation.

The AA composition based features include the composition of the 20 AA types in the input sequence, the composition of the residues of certain AA type in a given predicted secondary structure conformation, the composition of the residues of certain AA type which are either buried or exposed based on different RSA cutoffs, and the composition of the 400 dipeptide types (see Table 2).

**Table 2:** Comparison of the prediction performance of the Gaussian naïve Bayes (GNB)-based wrapper, logistic regression (LogR)-based wrapper, decision tree (DT)-based wrapper, k-nearest neighbor (KNN)-based wrapper, and two support vector machine (SVM)-based wrappers with the RBF and polynomial kernels (denoted as SVM-RBF and SVM-Poly respectively).

Wrapper method	Five-fold CV (average of 10 runs)				Jackknife test			
	Sen	Spe	Acc	MCC	Sen	Spe	Acc	MCC
GNB	0.815±0.010	0.767±0.009	**0.791**±0.007	**0.583**±0.014	0.828	**0.781**	**0.805**	**0.610**
DT	0.716±0.019	0.704±0.025	0.710±0.011	0.421±0.021	0.684	0.700	0.692	0.384
LogR	0.801±0.008	0.699±0.005	0.750±0.006	0.502±0.012	0.805	0.704	0.754	0.511
KNN	0.716±0.015	**0.770**±0.010	0.743±0.008	0.487±0.016	0.721	0.771	0.746	0.492
SVM-Poly	**0.867**±0.008	0.668±0.011	0.768±0.009	0.547±0.019	**0.855**	0.687	0.771	0.550
SVM-RBF	0.830±0.013	0.746±0.006	0.788±0.008	0.578±0.016	0.848	0.754	0.801	0.605

Note: The CV tests were based on ten runs and the averages and the standard deviations are shown. The highest values are shown in bold.

PSSM generated by PSI-BLAST has been widely used to represent the evolutionary information of a protein sequence, which was proved to be highly effective in a variety of prediction areas in protein structure bioinformatics, including the prediction of DNA-binding proteins [20], [23] and sites [43], function sites [41], [44], contact map [45], [46], disordered region [47], domain boundary [48], [49], solvent accessibility [37], to name just a few. The PSSM is a *L*×20 matrix, where *L* is the length of the protein sequence and 20 is the number of amino acid types. The score values are first normalized by using the following standard logistic function:
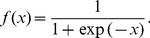
(1)


Next, we computed the average score of the residues with respect to the column of certain AA type, the average score of the residues of certain AA type with respect to the column of some AA type, the percentile value of the PSSM scores along with the column of certain AA type according to percent thresholds, and auto-correlation coefficient (AutoCC) of scores along with the column of certain AA type according to various lag values. The percent thresholds for the percentile statistics are set to be {0, 25, 50, 75, 100}. For a threshold *t*, the percentile statistics is the top (100−*t*)% value of scores in one column. Thus, threshold value 0 corresponds to the minimum score in one column of certain AA type, and threshold value 100 is actually associated with the maximum score in the column. The auto-correlation coefficient with certain lag can be calculated as follows:
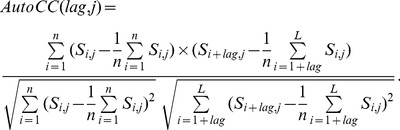
(2)where *n* is equal to *L-lag*, *L* is the length of the protein sequence, and *S_i,j_* is the PSSM score corresponding to the element in the *i-*th row and the *j*-th column in the matrix. The usage of AutoCC features was motivated by the wide-spread application of auto covariance to various fields of bioinformatics [50]–[52]. Here, AutoCC is actually a variant of auto covariance that the former is standardized between −1 and 1 while the later is not.

### 2.3. Random Forest and Gaussian Naïve Bayes

Random forest (RF) has been widely used for pattern recognition in bioinformatics [53]. It can provide not only the high prediction performance [8], [22] but also information on variable importance [53]–[55] for classification task. The algorithm of random forest is based on the ensemble of a large number of decision trees [56], where each tree gives a classification and the forest chooses the final classification having the most votes (over all the trees in the forest). In the most commonly used type of random forests, split selection is performed based on the so-called decrease of Gini impurity. In this study, the random forest is used to rank the features using Gini importance that is implemented with the machine learning platform scikit-learn [57].

Naïve Bayes (NB) is a set of supervised learning algorithms that apply Bayes’ theorem with the “naive” assumption of independence between every pair of features [58]. A NB classifier calculates the probability that a given instance (example) belongs to a certain class. Given an instance *X*, described by its feature vector (*x*_1_,…, *x_n_*), and a class target *y*, Bayes’ theorem allows us to express the conditional probability *P*(*y|X*) as a product of simpler probabilities using the naïve independence assumption:
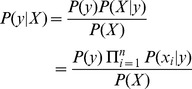
(3)


Since *P*(*X*) is constant for a given instance, the following rule is used to classify the sample:
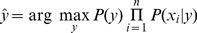
(4)


Maximum a posteriori (MAP) estimation is commonly used to estimate the parameters in the naïve Bayes model, including *P*(*y*) and *P*(*x_i_*|*y*); the former is the frequency of samples with class *y* in the training set. Moreover, Gaussian naïve Bayes (GNB) implements the classification by assuming the likelihood of the features to be Gaussian:
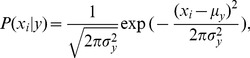
(5)where the parameters *σ_y_* and *µ_y_* are estimated by maximum likelihood. Due to its simplicity and being extremely fast compared to more sophisticated methods, GNB has been also widely applied to prediction problems in bioinformatics [59]–[61]. Here, GNB was used to train the prediction model of DNA-binding proteins and to perform the wrapper-based feature selection. On the other hand, our computational experiments in this work showed that GNB exhibited better performance than other classifiers, including logistic regression (LogR), decision tree (DT), k-nearest neighbor (KNN), and support vector machine (SVM). All of the machine learning methods were implemented in scikit-learn [57].

### 2.4. Performance Evaluation

Prediction performance is assessed using four quality indices including sensitivity (the ratio between the number of correct predictions for DNA-binding proteins and the total number of the actual DNA-binding proteins), specificity (the ratio between the number of correct predictions for non DNA-binding proteins and the total number of the actual non DNA-binding proteins), the overall accuracy, and Matthews correlation coefficient (MCC) [62]:
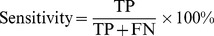


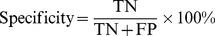


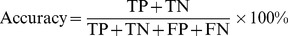


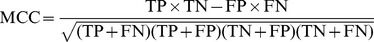
where true positives (TP) and true negatives (TN) correspond to correctly predicted DNA-binding and non DNA-binding proteins, respectively, false positives (FP) denote non DNA-binding proteins predicted as DNA-binding proteins, and false negatives (FN) denote DNA-binding proteins predicted as non DNA-binding proteins. The MCC measure ranges between −1 and 1, where −1 corresponds to all incorrect predictions, 0 to random predictions, and 1 to all correct predictions.

The performance is tested using *n*-fold cross validation (*n*CV) with multiple runs (to improve validity of the results) on PDB594 dataset. In the *n*CV, chains are randomly divided into *n* subsets with the same numbers of sequences, and the test is repeated *n* times, each time using one subset to test the prediction model and the remaining *n*−1 subsets to establish the model. Execution of one *n*CV is called a run and the *n* subsets for the run are named a seed. In the wrapper-based feature selection, we performed five-fold cross validation (5 CV), but we executed ten runs using ten different randomly created seeds. The sensitivity, specificity, accuracy and MCC are computed for each run and then averaged over the ten runs. The jackknife test (JKT), also called the leave-one-out test, is actually a *n*CV, where *n* is the total number of sequences in the dataset. We also performed the jackknife test but executed the only one run since each run would give the same result.

### 2.5. Feature Selection

**Figure 1:**
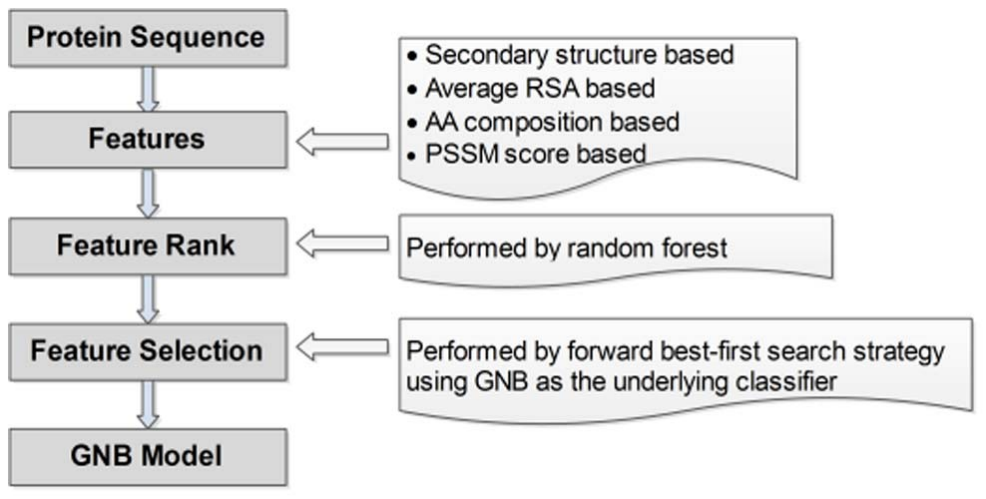
The flowchart of the proposed method.

The designed feature set is composed of 1486 descriptors. We performed feature selection since some of these features could be irrelevant to the prediction/characterization of DNA-binding proteins. Two stages were utilized in the wrapper based feature selection: (1) feature rank performed using random forest; (2) feature selection by forward best-first search combined with GNB classifier. In the first stage, top 300 features according to the Gini importance of random forest are selected. While in the second stage, feature selection is performed limited to this subset that is composed of 300 important features. The feature sets that lead to a higher average MCC are selected by performing the forward best-first search scheme. The computation of the MCC involves out-of-sample tests on the training set PDB594. More specifically, we execute ten random seeds of five-fold cross validation (5 CV) and use the average MCC to rank features. We start one feature that gives the largest MCC and then add the second feature (among the remaining 299 features) which results in the best average MCC. This is performed incrementally until adding an additional feature without obvious average MCC improvement. Figure 1 shows the flowchart of the proposed method.

In addition, other machine learning methods including LogR, DT, KNN and SVM are also applied to the above feature selection for a comparison. However, KNN needs set the number of neighbors, and the SVM classifiers require parametrization of the complexity constant C and the kernel function. The number of neighbors for KNN was limited to the set {5, 7, 9, 11, 13}. For each step of the above feature selection in which one feature was added into the previous selected feature set, KNN was performed over the all allowable numbers of neighbors and the one with the highest prediction performance was kept. For SVM, we consider two kernel types, radial basis function (RBF) *K*(*x_i_*,*x_j_*) = exp(−γ||*x_i_*−*x_j_*||^2^) where γ is the width of the RBF function, and polynomial *K*(*x_i_*,*x_j_*) = (*x_i·_x_j_*)*^d^* where *d* is the degree. When *d* = 1, the polynomial *K*(*x_i_*,*x_j_*) = (*x_i·_x_j_*) is actually the linear kernel. The SVM classifiers with these two types of kernels are denoted as SVM-RBF and SVM-Poly, respectively. We performed the grid search to optimize the parameters of SVM classifiers. For the RBF kernel, *C* = {2^−3^, 2^−2^, …, 2^2^, 2^3^} and γ = {2^−3^, 2^−2^, …, 2^2^, 2^3^}, and for polynomial kernel *C* = {2^−3^, 2^−2^, …, 2^2^, 2^3^} and *d* = {1, 2, 3}. The parameterization is performed again each time when an additional feature is added to the set of the selected features.

## Results

### 3.1. Performance of the Proposed Method

**Figure 2:**
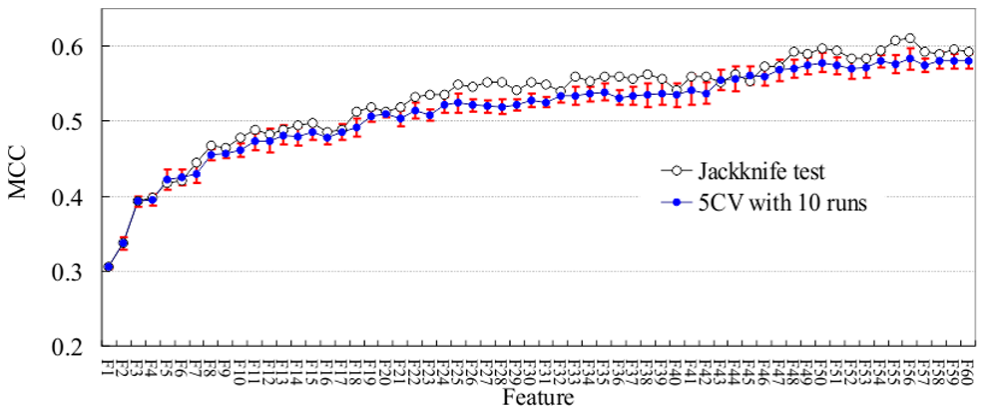
The improvement of MCC values (*y* axis) along with the increasing number of selected features (*x* axis) for the performed wrapper based feature selection. A forward, best-first search was executed using both 10 5 CV runs and jackknife tests on the PDB594 dataset. The standard deviations of MCC values for the case of 5 CV with 10 runs are shown using error bar.

The proposed method, called DBPPred, was implemented by ranking features using random forest algorithm and selecting features using forward best-first search strategy based on Gaussian naïve Bayes. Total of 300 features according to the feature rank were input to the subsequent feature selection, and each step in the forward best-first search by adding one remaining feature was performed based on 5 CV with 10 runs. Therefore, the results including sensitivity, specificity, accuracy and MCC were averaged over the ten runs, and their standard deviations were also reported. Figure 2 shows the improvement of MCC values along with the increasing number of selected features in the procedure of the forward, best-first search that was executed using 5 CV with 10 runs. The results from jackknife tests using the ranked features derived by the feature selection based on 5 CV with 10 runs were also shown in the figure. It can be observed that when the number of features is 56, the corresponding average MCC value based on 5 CV with 10 runs achieves the highest. Meanwhile, the MCC value derived by Jackknife test is also the highest.

Thus, the final feature set determined by the proposed method is composed of 56 features. The corresponding average sensitivity, specificity, accuracy and MCC values are 0.815, 0.767, 0.791 and 0.583, respectively, for 5 CV with 10 runs, and are 0.828, 0.781, 0.805 and 0.610, respectively, for jackknife test. Before the overall MCC peak achieved with 56 features, the procedure of feature selection provided in general improvement of MCC with the increasing number of selected features, however, the MCC value decreases a little bit when adding certain feature, such as the 16*^th^* feature and the 21^st^ feature. We emphasize that the combination of all selected features contribute to the final improvement on MCC value.

### 3.2. Comparison with Several Machine Learning Methods

Apart from GNB, several classifiers including DT, LogR, KNN, SVM-Poly and SVM-RBF were also applied to the feature selection procedure of the proposed method for a comparison. Table 2 lists the prediction performance of considered methods according to their MCC peaks achieved that are similar to the case of GNB in Figure 2. The results for 5 CV with 10 runs and Jackknife test are both reported. As shown in Table 2, for the case of 5 CV with 10 runs, the SVM-Poly based wrapper generates the highest average sensitivity of 0.867, and the KNN based wrapper yields the best average specificity of 0.770 although it is very close to the specificity of 0.767 achieved by GNB based wrapper. However, GNB based wrapper yields the highest average accuracy of 0.791 and average MCC of 0.583 and also provides better balance between sensitivity and specificity than SVM-Poly based wrapper. Similarly for the case of Jackknife test, SVM-Poly based wrapper outputs the highest sensitivity of 0.855, while GNB based wrapper yields the highest specificity of 0.781, accuracy of 0.805, and MCC of 0.610. It should be noted that the performance of SVM-RBF based wrapper is very close to the GNB based wrapper. Due to the simplicity of GNB when compared with SVM, GNB was finally determined as the underlying method.

### 3.3. Comparison of Independent Tests with Existing Methods

The independent dataset PDB186 was used to validate the quality of predictions for sequences that share low identity (<25%) with the training set. We performed blind test on PDB186 using the GNB model that was trained on the entire PDB594 dataset. We also compared the predictions of the proposed DBPPred on PDB186 with those of several relevant existing methods that have web server or standalone version concerning the sequence based predictions of DNA binding proteins. These methods include iDNA-Prot [8], DNA-Prot [22], DNAbinder [23], DNABIND [24], and DBD-Threader [19], to our best knowledge.

Table 3 shows the performance comparison of the proposed DBPPred with the five existing methods based on the PDB186 dataset. As shown in the table, the proposed DBPPred has the highest sensitivity of 0.796, accuracy of 0.769, and MCC of 0.538, and the secondly highest specificity of 0.742. The independent predictions of DBPPred are improved by accuracy of 9.2% and MCC of 0.183 when compared with the remaining best method, i.e. DBDBIND. The next method is iDNA-Prot, whose performance is very close to DBDBIND. DNA-Prot and DNAbinder are two close methods that have lower prediction quality than DBDBIND and iDNA-Prot. DBD-Threader was performed with the lowest accuracy of all considered methods. More specially, DBD-Threader achieved the lowest sensitivity of 0.237 and the highest specificity of 0.957, which implies that this method remarkably tends to predict a query protein as non DNA-binding whatever it is actually DNA-binding or non DNA-binding. As a result, DBD-Threader yields generates the lowest accuracy of 0.597. The reason may be due to the fact that DBD-Threader is actually a threading based method that requires a template library of DNA-binding proteins [19]. However, the size of the template library may be not large enough.

**Table 3:** Comparison of DBPPred with the existing methods based on independent blind tests on the same dataset PDB186.

Method	Reference	Sensitivity	Specificity	Accuracy	MCC	AUC
DBPPred	This work	**0.796**	0.742	**0.769**	**0.538**	**0.791**
iDNA-Prot	[8]	0.677	0.667	0.672	0.344	N/A
DNA-Prot	[22]	0.699	0.538	0.618	0.240	N/A
DNAbinder	[23]	0.570	0.645	0.608	0.216	0.607
DNABIND	[24]	0.667	0.688	0.677	0.355	0.694
DBD-Threader	[19]	0.237	**0.957**	0.597	0.279	N/A

N/A means that the data are not available.

**Figure 3:**
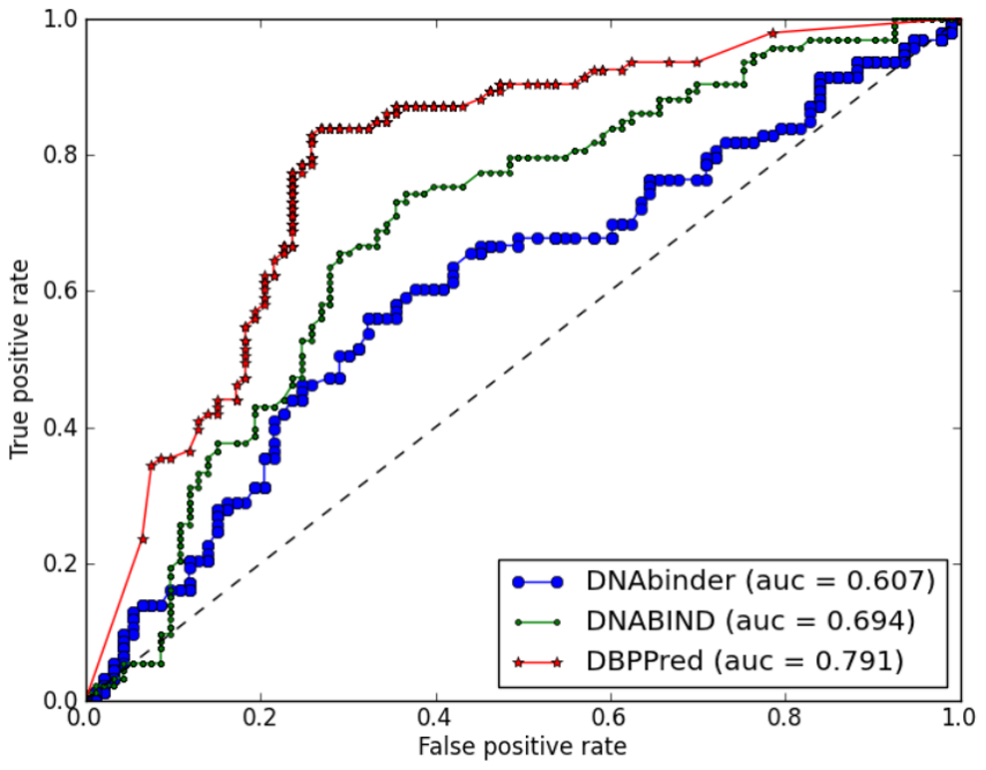
ROC curves for the predictions of DNA-binding proteins on the PDB186 dataset. We compare the predictions of DBPPred with DNABIND and DNAbinder that provide real-value outputs.

Moreover, two methods, DNAbinder and DNABIND, provide real-value outputs, which can be used to plot Receiver Operating Characteristic (ROC) [63] curve. We performed ROC analysis to further compare the prediction performance of the proposed method DBDPred, DNAbinder and DNABIND. The ROC curve shows the relation between true positive rate (sensitivity) and false positive rate (1-specificity) for each threshold of the real-value outputs. Figure 3 shows the ROC curves of the proposed DBPPred base on GNB, the DNAbinder based on SVM, and the DNABIND based on LogR. The areas under the ROC curves (AUCs), which quantify the overall performance independently of the threshold values, equal 0.791 for DBPPred, 0.607 for DNAbinder, and 0.694 for DNABIND. This indicates that the proposed DBPPred outperforms DNAbinder and DNABIND. The prediction results of all methods in Table 3 as well as the real-value outputs of the proposed DBPPred, DNAbinder and DNABIND are listed in Information S1.

Table 4 lists the false positive rates of the proposed DBPPred, iDNA-Prot, DNA-Prot, DNAbinder and DNABIND performed on several non-DNA binding protein datasets, NDBP4025, RB174, RB256 and RB430. We did not include the result of DBD-Threader into the table, since its prediction output probably tends to be negative (i.e. non-DNA binding protein) and the server is not friendly for large number of sequences. As shown in the table, DBPPred yields the smallest false positive rate of 0.254 (i.e. the specificity is 0.746) when compared with other methods including iDNA-Prot, DNA-Prot, DNAbinder and DNABIND, which achieve the false positive rates of 0.310, 0.354, 0.325, and 0.299, respectively, based on the NDBP4025 dataset. The results of all methods in Table 4 based on the dataset NDBP4025 are close to the specificity values derived from the independent tests on PDB186. In summary, DBPPred provides improved predictions of DNA-binding proteins with a balance of sensitivity and specificity. The prediction results of all methods in Table 4 performed on NDBP4025 dataset are listed in Information S2.

**Table 4:** List of false positive rates of the proposed DBPPred and the existing iDNA-Prot, DNA-Prot, DNAbinder and DNABIND on datasets NDBP4025, RB174, RB256 and RB430.

Method	False positive rate			
	NDBP4025	RB174	RB256	RB430
DBPPred	0.254	0.534	0.527	0.530
iDNA-Prot	0.310	0.483	0.559	0.528
DNA-Prot	0.354	0.713	0.703	0.707
DNAbinder	0.325	0.672	0.652	0.660
DNABIND	0.299	0.741	0.727	0.733

In case of RNA-binding proteins, as shown in Table 4, all methods show the limited ability to distinguish between DNA-binding and RNA-binding proteins. For the results performed on the three datasets RB174, RB256 and RB430, the smallest false positive rate achieved by iDNA-Prot based on RB174 dataset is 0.483, which is far from the largest false positive rate of 0.354 achieved by DNA-Prot based on NDBP4025 dataset. However, the false positive rate of the proposed DBPPred (0.530) is comparable with iDNA-Prot (0.528) and is smaller than those of DNA-Prot (0.707), DNAbinder (0.660) and DNABIND (0.733) based on the RB430 dataset. Specifically, DBPPred has larger false positive rate on RB174 and smaller false positive rate on RB256 when compared with iDNA-Prot, resulting in the comparable results between DBPPred and iDNA-Prot based on the union of RB174 and RB256, i.e. RB430. The prediction results of all methods in Table 4 performed on the two datasets RB174 and RB256 are listed in Information S3.

We conclude that the proposed DBPPred provides favorable results, which should allow for building a well-performing DNA-binding protein predictor. Additionally, a standalone software of the proposed model that predicts the DNA-binding protein is provided as Software S1.

### 3.4. Analysis of Selected Features

Table 5 lists the 56 features that are selected in the proposed DBPPred. The features in the table have been already ordered according to the feature addition procedure in the forward best-first search strategy. Of all selected features, 38 out of 56 features are PSSM score based, 17 out of 56 features are AA composition based, and one out of 56 features is average RSA based. The majority (38/56 = 67.9%) of the selected features are PSSM score based, showing that the evolutionary information generated by PSI-BLAST plays important roles in the prediction of DNA-binding proteins.

**Table 5:** The mean values of the selected 56 features and the P values that quantify significance of the differences between DNA-binding and non DNA-binding proteins for PDB594 dataset.

Feature	Category	Mean±std		P-value
		DNA-binding	Non DNA-binding	
Pscore_AA_Q__P_75_	PSSM score based	0.696±0.095	0.626±0.124	<10^−3^
AvePscore_AA_Y__Res_K_	PSSM score based	0.160±0.078	0.207±0.093	<10^−3^
AveRSA_Res_G_	Average RSA based	0.310±0.076	0.271±0.061	<10^−3^
AvePscore_AA_P_	PSSM score based	0.232±0.049	0.255±0.058	<10^−3^
DIC_Res_KS_	AA composition based	0.005±0.006	0.004±0.005	0.019
AvePscore_AA_R__Res_G_	PSSM score based	0.227±0.124	0.224±0.096	0.765
AutoCC_AA_N_ _Lag_7_	PSSM score based	−0.014±0.106	0.010±0.095	0.003
AvePscore_AA_G__Res_R_	PSSM score based	0.170±0.094	0.207±0.103	<10^−3^
AvePscore_AA_L_	PSSM score based	0.323±0.057	0.329±0.048	0.117
AvePscore_AA_K__Res_G_	PSSM score based	0.276±0.122	0.266±0.100	0.272
AvePscore_AA_Q_	PSSM score based	0.422±0.040	0.396±0.051	<10^−3^
AvePscore_AA_R__Res_M_	PSSM score based	0.250±0.164	0.229±0.124	0.080
AAC_Res_R__Ex_0.2_	AA composition based	0.092±0.043	0.080±0.037	<10^−3^
AvePscore_AA_T_	PSSM score based	0.377±0.037	0.387±0.040	0.001
AAC_Res_D__Bu_0.3_	AA composition based	0.018±0.019	0.024±0.016	<10^−3^
AAC_Res_V__Ex_0.1_	AA composition based	0.039±0.022	0.045±0.019	<10^−3^
AutoCC_AA_R_ _Lag_7_	PSSM score based	0.008±0.091	0.031±0.100	0.004
AAC_Res_N_	AA composition based	0.038±0.021	0.043±0.022	0.003
AvePscore_AA_C__Res_N_	PSSM score based	0.141±0.140	0.136±0.096	0.644
AutoCC_AA_I_ _Lag_7_	PSSM score based	−0.015±0.092	0.009±0.097	0.002
AAC_Res_D__Bu_0.2_	AA composition based	0.012±0.018	0.016±0.015	0.004
AvePscore_AA_G__Res_K_	PSSM score based	0.197±0.090	0.232±0.107	<10^−3^
AvePscore_AA_R__Res_E_	PSSM score based	0.493±0.100	0.478±0.085	0.044
AutoCC_AA_I_ _Lag_8_	PSSM score based	−0.010±0.105	−0.005±0.076	0.511
AAC_Res_A__Ex_0.1_	AA composition based	0.062±0.035	0.068±0.037	0.04
AAC_Res_T__Ex_0.3_	AA composition based	0.044±0.028	0.055±0.033	<10^−3^
AutoCC_AA_P_ _Lag_7_	PSSM score based	−0.011±0.110	0.021±0.096	<10^−3^
AutoCC_AA_E_ _Lag_4_	PSSM score based	0.106±0.120	0.100±0.125	0.524
AAC_Res_E__Ex_0.1_	AA composition based	0.093±0.032	0.088±0.035	0.049
AutoCC_AA_I_ _Lag_3_	PSSM score based	0.029±0.092	0.007±0.085	0.003
AutoCC_AA_K_ _Lag_7_	PSSM score based	0.021±0.101	0.041±0.105	0.018
AvePscore_AA_R_ _Res_R_	PSSM score based	0.930±0.086	0.892±0.121	<10^−3^
AutoCC_AA_R__Lag_9_	PSSM score based	−0.021±0.110	−0.007±0.079	0.084
AAC_Res_L__Bu_0.5_	AA composition based	0.111±0.038	0.102±0.031	0.003
AvePscore_AA_R__Res_W_	PSSM score based	0.124±0.200	0.167±0.202	0.008
Pscore_AA_R_ _P_75_	PSSM score based	0.661±0.154	0.584±0.152	<10^−3^
AAC_Res_T__Ex_0.4_	AA composition based	0.035±0.033	0.044±0.036	0.002
AvePscore_AA_I__Res_D_	PSSM score based	0.089±0.065	0.108±0.059	<10^−3^
AvePscore_AA_N__Res_R_	PSSM score based	0.402±0.106	0.417±0.108	0.105
AutoCC_AA_V_ _Lag_8_	PSSM score based	−0.015±0.099	−0.009±0.077	0.381
AvePscore_AA_H__Res_W_	PSSM score based	0.145±0.202	0.214±0.211	<10^−3^
AvePscore_AA_R_	PSSM score based	0.387±0.055	0.357±0.055	<10^−3^
AvePscore_AA_W__Res_T_	PSSM score based	0.094±0.083	0.148±0.110	<10^−3^
AAC_Res_N__Bu_0.2_	AA composition based	0.011±0.016	0.015±0.015	0.001
AutoCC_AA_I_ _Lag_4_	PSSM score based	0.014±0.106	−0.010±0.099	0.004
AvePscore_AA_E_	PSSM score based	0.407±0.043	0.391±0.055	<10^−3^
DIP_Res_DL_	AA composition based	0.005±0.006	0.005±0.005	0.687
AvePscore_AA_N__Res_I_	PSSM score based	0.104±0.085	0.129±0.080	<10^−3^
AutoCC_AA_C__Lag_7_	PSSM score based	−0.002±0.103	0.020±0.082	0.003
AutoCC_AA_L_ _Lag_7_	PSSM score based	−0.005±0.101	0.016±0.103	0.012
AvePscore_AA_I__Res_A_	PSSM score based	0.285±0.100	0.287±0.098	0.745
AAC_Res_A__Bu_0.2_	AA composition based	0.096±0.056	0.102±0.050	0.158
AAC_Res_E_	AA composition based	0.074±0.025	0.069±0.027	0.009
AutoCC_AA_T__Lag_2_	PSSM score based	0.011±0.101	0.050±0.090	<10^−3^
AAC_Res_T__Ex_0.2_	AA composition based	0.052±0.027	0.061±0.029	<10^−3^
AAC_Res_C__SS_C_	AA composition based	0.011±0.023	0.016±0.024	0.019

Furthermore, we investigate statistical significance of the differences of these feature values between the DNA-binding and non DNA-binding proteins on the PDB594 dataset. Table 5 gives the *P* values of two-sided *t* tests. It can be observed that if the statistically significant difference (SSD) between DNA-binding and non DNA-binding proteins is at 0.05 level, 43 out of 56 (43/56 = 76.8%) features have *P* values less than 0.05, and thus their differences of the feature values of DNA-binding and non DNA-binding proteins are statistically significant. As expected, the results confirm that the majority of the selected features by the proposed method have statistically significant differences between the DNA-binding and non DNA-binding proteins.

It can be observed that the secondary structure based features are not selected in the final model. However, we strengthen that the high quality of the proposed method is attributed to the combination of the selected features. In addition, an alternative reason may be due to that the secondary structures were predicted from evolutionary information in SPINE-X program. When a number of features associated with PSSM scores were already selected, the predicted SS based features contributed no more improvement to the prediction of DNA-binding proteins, and then they were not selected.

## Conclusion

In this work, we proposed a new method, called DBPPred, for the prediction of the DNA-binding proteins, by performing the feature rank using random forest and the wrapper-based feature selection using forward best-first search strategy and Gaussian naïve Bayes as the underlying classifier. The features comprise information from the primary sequence, the predicted secondary structure, the predicted relative solvent accessibility, and the position specific scoring matrix. The proposed method using GNB as the underlying classifier was compared with other five classifiers having the same cross validation procedures, including decision tree, logistic regression, k-nearest neighbor, SVM with polynomial kernel, and SVM with RBF kernel. As a result, the proposed DBPPred performs the best according to the five-fold cross validation with ten runs on PDB594 dataset. Moreover, independent tests of the proposed DBPPred, which was trained on the entire dataset PDB594, and other five existing methods including iDNA-Prot, DNA-Prot, DNAbinder, DNABIND and DBD-Threader were performed on the PDB186 dataset, resulting in that DBPPred yielded the highest prediction quality. All of the experimental results, including additional tests on purely the non-DNA binding protein dataset NDBP2045 and the RNA-binding protein dataset RB430, indicate that the proposed DBPPred may be an alternative perspective predictor for large-scale determination of DNA-binding proteins.

## Supporting Information

Dataset S1 **The PDB594 dataset.** This dataset was used for training. The file includes the PDB IDs, the deposition dates, the target values denoting that the proteins are DNA-binding or not, and the corresponding primary sequences.
(TXT)

Dataset S2 **The PDB186 dataset.** This dataset was used for independent blind test. The file includes the PDB IDs, the deposition dates, the target values denoting that the proteins are DNA-binding or not, and the corresponding primary sequences.
(TXT)

Information S1 **The prediction results of the proposed DBPPred, iDNA-Prot, DNA-Prot, DNAbinder, DNABIND and DBD-Threader on PDB186 dataset.** The file lists the actual target value and the predicted values of the existing methods for each sequence in PDB186 dataset. The real-value outputs of three methods, DBPPred, DNAbinder and DNABIND, are also provided.
(TXT)

Information S2 **The prediction results of the proposed DBPPred, iDNA-Prot, DNA-Prot, DNAbinder and DNABIND on NDBP4025 dataset.** The file lists the predicted values of the existing methods for each sequence in NDBP4025 dataset.
(TXT)

Information S3 **The prediction results of the proposed DBPPred, iDNA-Prot, DNA-Prot, DNAbinder and DNABIND on datasets RB174 and RB256.** The file lists the predicted values of the existing methods for each sequence in RB174 and RB256 dataset.
(TXT)

Software S1 **The program of the proposed DBPPred model that predicts the DNA-binding proteins.** This ZIP file contains python scripts together with instruction how to run it.
(ZIP)

## References

[1] Sarai A, Kono H (2005) Protein-DNA recognition patterns and predictions. Annu Rev Biophys Biomol Struct 34: 379–398 10.1146/annurev.biophys.34.040204.144537 15869395

[2] Liu LA, Bradley P (2012) Atomistic modeling of protein-DNA interaction specificity: progress and applications. Curr Opin Struct Biol 22: 397–405 10.1016/j.sbi.2012.06.002 22796087 PMC3425445

[3] Langlois RE, Lu H (2010) Boosting the prediction and understanding of DNA-binding domains from sequence. Nucleic Acids Res 38: 3149–3158 10.1093/nar/gkq061 20156993 PMC2879530

[4] Cajone F, Salina M, Benelli-Zazzera A (1989) 4-Hydroxynonenal induces a DNA-binding protein similar to the heat-shock factor. Biochem J 262: 977–979. 2590181 10.1042/bj2620977 PMC1133369

[5] Buck MJ, Lieb JD (2004) ChIP-chip: considerations for the design, analysis, and application of genome-wide chromatin immunoprecipitation experiments. Genomics 83: 349–360. 14986705 10.1016/j.ygeno.2003.11.004

[6] Freeman K, Gwadz M, Shore D (1995) Molecular and genetic analysis of the toxic effect of RAP1 overexpression in yeast. Genetics 141: 1253–1262. 8601471 10.1093/genetics/141.4.1253 PMC1206864

[7] Chou CC, Lin TW, Chen CY, Wang AHJ (2003) Crystal structure of the hyperthermophilic archaeal DNA-binding protein Sso10b2 at a resolution of 1.85 Angstroms. J Bacteriol 185: 4066–4073. 12837780 10.1128/JB.185.14.4066-4073.2003 PMC164892

[8] Lin WZ, Fang JA, Xiao X, Chou KC (2011) iDNA-Prot: identification of DNA binding proteins using random forest with grey model. PloS One 6: e24756 10.1371/journal.pone.0024756 21935457 PMC3174210

[9] Stawiski EW, Gregoret LM, Mandel-Gutfreund Y (2003) Annotating nucleic acid-binding function based on protein structure. J Mol Biol 326: 1065–1079. 12589754 10.1016/s0022-2836(03)00031-7

[10] Ahmad S, Sarai A (2004) Moment-based prediction of DNA-binding proteins. J Mol Biol 341: 65–71 10.1016/j.jmb.2004.05.058 15312763

[11] Gao M, Skolnick J (2008) DBD-Hunter: a knowledge-based method for the prediction of DNA-protein interactions. Nucleic Acids Res 36: 3978–3992 10.1093/nar/gkn332 18515839 PMC2475642

[12] Zhao H, Yang Y, Zhou Y (2010) Structure-based prediction of DNA-binding proteins by structural alignment and a volume-fraction corrected DFIRE-based energy function. Bioinforma Oxf Engl 26: 1857–1863 10.1093/bioinformatics/btq295 PMC2905551 20525822

[13] Nimrod G, Szilágyi A, Leslie C, Ben-Tal N (2009) Identification of DNA-binding proteins using structural, electrostatic and evolutionary features. J Mol Biol 387: 1040–1053 10.1016/j.jmb.2009.02.023 19233205 PMC2726711

[14] Nimrod G, Schushan M, Szilágyi A, Leslie C, Ben-Tal N (2010) iDBPs: a web server for the identification of DNA binding proteins. Bioinformatics 26: 692–693 10.1093/bioinformatics/btq019 20089514 PMC2828122

[15] Zhou W, Yan H (2011) Prediction of DNA-binding protein based on statistical and geometric features and support vector machines. Proteome Sci 9 Suppl 1 S1 10.1186/1477-5956-9-S1-S1 22166014 PMC3289070

[16] Szabóová A, Kuželka O, Zelezný F, Tolar J (2012) Prediction of DNA-binding propensity of proteins by the ball-histogram method using automatic template search. BMC Bioinformatics 13 Suppl 10 S3 10.1186/1471-2105-13-S10-S3 PMC3382442 22759427

[17] Bhardwaj N, Langlois RE, Zhao G, Lu H (2005) Kernel-based machine learning protocol for predicting DNA-binding proteins. Nucleic Acids Res 33: 6486–6493 10.1093/nar/gki949 16284202 PMC1283538

[18] Bhardwaj N, Lu H (2007) Residue-level prediction of DNA-binding sites and its application on DNA-binding protein predictions. FEBS Lett 581: 1058–1066 10.1016/j.febslet.2007.01.086 17316627 PMC1993824

[19] Gao M, Skolnick J (2009) A threading-based method for the prediction of DNA-binding proteins with application to the human genome. PLoS Comput Biol 5: e1000567 10.1371/journal.pcbi.1000567 19911048 PMC2770119

[20] Zou C, Gong J, Li H (2013) An improved sequence based prediction protocol for DNA-binding proteins using SVM and comprehensive feature analysis. BMC Bioinformatics 14: 90 10.1186/1471-2105-14-90 23497329 PMC3602657

[21] Huang HL, Lin IC, Liou YF, Tsai CT, Hsu KT, et al (2011) Predicting and analyzing DNA-binding domains using a systematic approach to identifying a set of informative physicochemical and biochemical properties. BMC Bioinformatics 12 Suppl 1 S47 10.1186/1471-2105-12-S1-S47 21342579 PMC3044304

[22] Kumar KK, Pugalenthi G, Suganthan PN (2009) DNA-Prot: identification of DNA binding proteins from protein sequence information using random forest. J Biomol Struct Dyn 26: 679–686. 19385697 10.1080/07391102.2009.10507281

[23] Kumar M, Gromiha MM, Raghava GPS (2007) Identification of DNA-binding proteins using support vector machines and evolutionary profiles. BMC Bioinformatics 8: 463 10.1186/1471-2105-8-463 18042272 PMC2216048

[24] Szilágyi A, Skolnick J (2006) Efficient Prediction of Nucleic Acid Binding Function from Low-resolution Protein Structures. J Mol Biol 358: 922–933 10.1016/j.jmb.2006.02.053 16551468

[25] Fang Y, Guo Y, Feng Y, Li M (2008) Predicting DNA-binding proteins: approached from Chou’s pseudo amino acid composition and other specific sequence features. Amino Acids 34: 103–109 10.1007/s00726-007-0568-2 17624492

[26] Nanni L, Lumini A (2008) Combing ontologies and dipeptide composition for predicting DNA-binding proteins. Amino Acids 34: 635–641 10.1007/s00726-007-0016-3 18175049

[27] Nanni L, Lumini A (2009) An ensemble of reduced alphabets with protein encoding based on grouped weight for predicting DNA-binding proteins. Amino Acids 36: 167–175 10.1007/s00726-008-0044-7 18288459

[28] Yu X, Cao J, Cai Y, Shi T, Li Y (2006) Predicting rRNA-, RNA-, and DNA-binding proteins from primary structure with support vector machines. J Theor Biol 240: 175–184 10.1016/j.jtbi.2005.09.018 16274699

[29] Shao X, Tian Y, Wu L, Wang Y, Jing L, et al (2009) Predicting DNA- and RNA-binding proteins from sequences with kernel methods. J Theor Biol 258: 289–293 10.1016/j.jtbi.2009.01.024 19490865

[30] Cai Y, Lin SL (2003) Support vector machines for predicting rRNA-, RNA-, and DNA-binding proteins from amino acid sequence. Biochim Biophys Acta 1648: 127–133. 12758155 10.1016/s1570-9639(03)00112-2

[31] Berman HM, Westbrook J, Feng Z, Gilliland G, Bhat TN, et al (2000) The Protein Data Bank. Nucleic Acids Res 28: 235–242. 10592235 10.1093/nar/28.1.235 PMC102472

[32] Altschul SF, Madden TL, Schäffer AA, Zhang J, Zhang Z, et al (1997) Gapped BLAST and PSI-BLAST: a new generation of protein database search programs. Nucleic Acids Res 25: 3389–3402. 9254694 10.1093/nar/25.17.3389 PMC146917

[33] Zhao H, Yang Y, Zhou Y (2011) Structure-based prediction of RNA-binding domains and RNA-binding sites and application to structural genomics targets. Nucleic Acids Res 39: 3017–3025 10.1093/nar/gkq1266 21183467 PMC3082898

[34] Zhao H, Yang Y, Zhou Y (2011) Highly accurate and high-resolution function prediction of RNA binding proteins by fold recognition and binding affinity prediction. RNA Biol 8: 988–996 10.4161/rna.8.6.17813 21955494 PMC3360076

[35] Shazman S, Mandel-Gutfreund Y (2008) Classifying RNA-binding proteins based on electrostatic properties. PLoS Comput Biol 4: e1000146 10.1371/journal.pcbi.1000146 18716674 PMC2518515

[36] Han L, Suzek TO, Wang Y, Bryant SH (2010) The Text-mining based PubChem Bioassay neighboring analysis. BMC Bioinformatics 11: 549 10.1186/1471-2105-11-549 21059237 PMC3098095

[37] Faraggi E, Zhang T, Yang Y, Kurgan L, Zhou Y (2012) SPINE X: improving protein secondary structure prediction by multistep learning coupled with prediction of solvent accessible surface area and backbone torsion angles. J Comput Chem 33: 259–267 10.1002/jcc.21968 22045506 PMC3240697

[38] Ivankov DN, Finkelstein AV (2004) Prediction of protein folding rates from the amino acid sequence-predicted secondary structure. Proc Natl Acad Sci U S A 101: 8942–8944 10.1073/pnas.0402659101 15184682 PMC428451

[39] Zhang H, Zhang T, Gao J, Ruan J, Shen S, et al (2012) Determination of protein folding kinetic types using sequence and predicted secondary structure and solvent accessibility. Amino Acids 42: 271–283 10.1007/s00726-010-0805-y 21082205

[40] Zhang T, Zhang H, Chen K, Ruan J, Shen S, et al (2010) Analysis and prediction of RNA-binding residues using sequence, evolutionary conservation, and predicted secondary structure and solvent accessibility. Curr Protein Pept Sci 11: 609–628. 20887256 10.2174/138920310794109193

[41] Zhang T, Zhang H, Chen K, Shen S, Ruan J, et al (2008) Accurate sequence-based prediction of catalytic residues. Bioinformatics 24: 2329–2338 10.1093/bioinformatics/btn433 18710875

[42] Ahmad S, Gromiha MM, Sarai A (2003) Real value prediction of solvent accessibility from amino acid sequence. Proteins 50: 629–635 10.1002/prot.10328 12577269

[43] Dey S, Pal A, Guharoy M, Sonavane S, Chakrabarti P (2012) Characterization and prediction of the binding site in DNA-binding proteins: improvement of accuracy by combining residue composition, evolutionary conservation and structural parameters. Nucleic Acids Res 40: 7150–7161 10.1093/nar/gks405 22641851 PMC3424558

[44] Walia RR, Caragea C, Lewis BA, Towfic FG, Terribilini M, et al (2012) Protein-RNA interface residue prediction using machine learning: an assessment of the state of the art. BMC Bioinformatics 13: 89 10.1186/1471-2105-13-89 22574904 PMC3490755

[45] Eickholt J, Cheng J (2012) Predicting protein residue-residue contacts using deep networks and boosting. Bioinformatics 28: 3066–3072 10.1093/bioinformatics/bts598 23047561 PMC3509494

[46] Tegge AN, Wang Z, Eickholt J, Cheng J (2009) NNcon: improved protein contact map prediction using 2D-recursive neural networks. Nucleic Acids Res 37: W515–518 10.1093/nar/gkp305 19420062 PMC2703959

[47] Zhang T, Faraggi E, Xue B, Dunker AK, Uversky VN, et al (2012) SPINE-D: accurate prediction of short and long disordered regions by a single neural-network based method. J Biomol Struct Dyn 29: 799–813. 22208280 10.1080/073911012010525022 PMC3297974

[48] Li BQ, Hu LL, Chen L, Feng KY, Cai YD, et al (2012) Prediction of Protein Domain with mRMR Feature Selection and Analysis. PLoS ONE 7. Available: http://www.ncbi.nlm.nih.gov/pmc/articles/PMC3376124/. Accessed 2013 July 10. 10.1371/journal.pone.0039308 PMC3376124 22720092

[49] Zhang X, Lu L, Song Q, Yang Q, Li D, et al (2013) DomHR: Accurately Identifying Domain Boundaries in Proteins Using a Hinge Region Strategy. PLoS ONE 8: e60559 10.1371/journal.pone.0060559 23593247 PMC3623903

[50] Guo Y, Li M, Lu M, Wen Z, Huang Z (2006) Predicting G-protein coupled receptors-G-protein coupling specificity based on autocross-covariance transform. Proteins 65: 55–60 10.1002/prot.21097 16865706

[51] Dong Q, Zhou S, Guan J (2009) A new taxonomy-based protein fold recognition approach based on autocross-covariance transformation. Bioinforma Oxf Engl 25: 2655–2662 10.1093/bioinformatics/btp500 19706744

[52] Guo Y, Yu L, Wen Z, Li M (2008) Using support vector machine combined with auto covariance to predict protein-protein interactions from protein sequences. Nucleic Acids Res 36: 3025–3030 10.1093/nar/gkn159 18390576 PMC2396404

[53] Touw WG, Bayjanov JR, Overmars L, Backus L, Boekhorst J, et al (2013) Data mining in the Life Sciences with Random Forest: a walk in the park or lost in the jungle? Brief Bioinform 14: 315–326 10.1093/bib/bbs034 22786785 PMC3659301

[54] Ebina T, Toh H, Kuroda Y (2011) DROP: an SVM domain linker predictor trained with optimal features selected by random forest. Bioinforma Oxf Engl 27: 487–494 10.1093/bioinformatics/btq700 21169376

[55] Boulesteix AL, Bender A, Lorenzo Bermejo J, Strobl C (2012) Random forest Gini importance favours SNPs with large minor allele frequency: impact, sources and recommendations. Brief Bioinform 13: 292–304 10.1093/bib/bbr053 21908865

[56] Breiman L (2001) Random Forests. Mach Learn 45: 5–32 10.1023/A:1010933404324

[57] Pedregosa F, Varoquaux G, Gramfort A, Michel V, Thirion B, et al (2011) Scikit-learn: Machine Learning in Python. J Mach Learn Res 12: 2825–2830.

[58] Mitchell TM (1997) Machine Learning. 1st edition. New York: McGraw-Hill.

[59] Cao J, Panetta R, Yue S, Steyaert A, Young-Bellido M, et al (2003) A naive Bayes model to predict coupling between seven transmembrane domain receptors and G-proteins. Bioinforma Oxf Engl 19: 234–240. 10.1093/bioinformatics/19.2.234 12538244

[60] Murakami Y, Mizuguchi K (2010) Applying the Naïve Bayes classifier with kernel density estimation to the prediction of protein-protein interaction sites. Bioinforma Oxf Engl 26: 1841–1848 10.1093/bioinformatics/btq302 20529890

[61] Raizada RDS, Lee YS (2013) Smoothness without Smoothing: Why Gaussian Naive Bayes Is Not Naive for Multi-Subject Searchlight Studies. PLoS ONE 8: e69566 10.1371/journal.pone.0069566 23922740 PMC3724912

[62] Matthews BW (1975) Comparison of the predicted and observed secondary structure of T4 phage lysozyme. Biochim Biophys Acta 405: 442–451. 1180967 10.1016/0005-2795(75)90109-9

[63] Sonego P, Kocsor A, Pongor S (2008) ROC analysis: applications to the classification of biological sequences and 3D structures. Brief Bioinform 9: 198–209 10.1093/bib/bbm064 18192302

